# Surgical management and outcome of medulloblastoma patients at Addis Ababa between 2010 and 2018

**DOI:** 10.3389/fonc.2025.1672757

**Published:** 2025-11-03

**Authors:** Yemisirach B. Akililu, Eric Bouffet, Mersha Abebe

**Affiliations:** ^1^ Neurosurgery Unit, Department of Surgery, College of Health Sciences, Addis Ababa University, Addis Ababa, Ethiopia; ^2^ The Hospital for Sick Children, University of Toronto, Toronto, ON, Canada

**Keywords:** medulloblastoma, surgery, morbidity, postoperative complications, low-income country

## Abstract

**Background:**

Medulloblastoma is the most common malignant central nervous system tumor in pediatric patients and is relatively rare in adults. Despite remarkable improvement in survival over recent decades, the high success rates in management documented in the Western world are not mirrored in middle- and low-income countries, due to various socio-economic factors and health-care disparities.

**Objective:**

The aim of this study was to assess surgical treatment outcome and prognostic factors in operated patients with medulloblastoma in Tikur Anbessa Specialized Hospital and Myungsung Christian Medical Center, the 2 largest neurosurgical units in Ethiopia.

**Methods:**

This retrospective study involved 27 patients (21 children and 6 adolescent or young adults) who underwent surgery for medulloblastoma between January 2010 and April 2018. A structured questionnaire was used to collect patients’ data and their treatment outcomes.

**Results:**

the 6 months and 1-year overall survival (OS) were 41% and 29% respectively, with a median survival time of 107 days (95% CI: 49,166). Median OS was 66 days (95% CI: 27,105), with 6 months and one-year OS rate of 24% for pediatric patients. Younger age at presentation, postoperative open EVD, longer Intensive Care Unit stay, lack of radiation treatment & postoperative complications were negative predictors of outcome.

**Conclusion:**

This experience underscores the high number of undiagnosed patients with medulloblastoma in Ethiopia and the poor outcome of patients who undergo active treatment. Standardization of treatment with surgery, craniospinal irradiation ± chemotherapy yields longer survival rates in both pediatric and adult patients.

## Introduction

Medulloblastoma is the most common central nervous system (CNS) malignant tumor in children. Modern literature highlights improvement in outcome in high income countries related to advances in diagnosis, multimodality treatment including surgery, radiotherapy and chemotherapy with 5-year overall survival (OS) rates of 80-85% for average risk patients and 60-70% in high-risk patients (1, 2). Nevertheless, this improvement is not well appreciated in low- and middle-income countries. This difference in outcomes could be ascribed to socio-cultural issues, demographic variations, health-care disparities, co-morbidities and resource constraints that result in delayed diagnosis and referral, lack of multidisciplinary care and limited resources for optimal management (3). The issue is compounded by a higher morbidity and mortality in the peri- operative setting (advanced stage at presentation, emergency surgery, higher risk of infection) as well as considerable delays in the initiation of post-operative adjuvant therapy (delay in referral, long waiting times for radiation therapy, prolonged course of radiotherapy, and relative lack of specialty centers and specialists as well), all of which could potentially contribute to inferior outcomes. Even with timely referral and available expertise, there is lack in uniformity of care resulting in variations in clinical practice (neurosurgical, pathology and chemo-radiotherapy approaches) and resultant outcomes.

Ethiopia is a low-income country according to the World Bank with a per capita gross national income of $1,020 (https://data.worldbank.org/country/ethiopia, accessed 29/9/2025). With 132 million people, it is the second most populous country in Africa after Nigeria, and the percentage of children < 14 years old is in the range of 40% (https://data.worldbank.org/indicator/SP.POP.0014.TO?locations=ET, accessed 29/9/2025). Access to care is limited due to low healthcare funding, limited well equipped health care facilities and for patients and families, high out-of-pocket expenditures despite the implementation of several reforms in healthcare financing over the last decades. Ethiopia’s health spending constituted 2.85% of the gross domestic product (GDP) in 2022 (https://data.worldbank.org/indicator/SH.XPD.CHEX.GD.ZS?locations=ET, accessed 10/07/2025), which is below the average 5.06% of WHO estimation for low-income countries (https://data.worldbank.org/indicator/SH.XPD.CHEX.GD.ZS?locations=XM, accessed 10/07/2025).

The Addis Ababa cancer registry reported an overall incidence rate of childhood cancer from 2012 to 2017 of 81.9 cases per million, which is 50% lower than rates reported in Europe and North America. CNS tumors represented only 3.3% of all malignancies in this registry (4). There has been only one report on CNS tumors on 86 children < 15 years of age seen at Tikur Anbessa Specialized Hospital Department of pediatrics from January 2014 to January 2019. (5). There is scant information on the surgical management of medulloblastoma patients in low-income countries, where limited resources may have a major impact on patients’ care. The aim of this report is to describe the surgical management, the postoperative complications, and the outcome and to assess prognostic factors in a series of consecutive patients operated with medulloblastoma. in 2 large tertiary hospitals at Addis Ababa. Ultimately, this work aimed to identify areas of focus for improved surgical and multidisciplinary management.

## Patients and methods

We conducted a hospital based retrospective study of medulloblastoma cases operated between January 1,2010 and April 30, 2018, at Tikur Anbessa Specialized Hospital (TASH) and Myungsung Christian Medical Center (MCM); two of the neurosurgical teaching hospitals in Addis Ababa, Ethiopia. Tikur Anbessa Specialized hospital is one of the oldest teaching hospitals in the country; in fact, is the only tertiary hospital where pediatrics brain tumors are being treated with proper neurosurgical intervention, chemotherapy, as well as radiotherapy. It is a referral center for patients with brain tumors across the country with 6 operating tables (6 slots allowed for elective per week) and 10 intensive care beds for all specialties (4 pediatric and 6 adult Intensive Care Unit (ICU) beds). Myungsung Christian Medical Center is a private comprehensive specialized hospital and teaching center as well, located 9.4 kilometers away from Tikur Anbessa Specialized hospital in the eastern part of Addis Ababa. These hospitals were selected for this particular study as they are 2 large neurosurgical units in the capital with a team of experienced anesthesiologists and intensive care units providing excellent continuity of postoperative care. In addition, the pathology and radiology departments are well established at these centers. However, due to lack of oncology unit at MCM hospital, patients need to be referred to TASH oncology department after surgical intervention and histopathologic confirmation of medulloblastoma for adjuvant treatment, i.e. radiotherapy and chemotherapy. As the TASH oncology unit was the only center where radiotherapy was being instituted in the whole country during this period, patients had long waiting lists before they could undergo postoperative radiation treatment.

The following data were collected for each patient: age, sex, clinical presentation, duration of presenting symptoms, preoperative performance status, risk stratification, presence of hydrocephalus, radiological characteristics, preoperative CSF diversion, extent of tumor resection, histological characteristics, postoperative complications, post operative radiotherapy and chemotherapy, outcome. Extent of resection was extrapolated from operation notes or immediate postoperative scan when available. The modified Chang staging system was used for risk classification. However, as most patients did not undergo a full staging, metastatic disease was not included in the staging criteria for patients without MRI scan of the spine or CSF cytology. The functional status of pediatric patients was assessed with the Pediatric Cerebral Performance Category Scale (PCPCS) (6) while the Karnofsky index was used for adolescent and adult patients.

### Statistical design

Data were entered and analyzed using Microsoft Excel Workbook and IBM SPSS Statistics 21. The descriptive statistics and frequencies were performed for all baseline characteristics of the study subjects. Results on continuous measurements were presented on mean +/- SD while categorical measurements were presented in number (%). Overall survival was calculated using KM curve and log rank test was used for comparisons. Differences were considered statistically significant for P values of <0.05.

Ethical approval for the study was obtained from the research committee of the Department of Surgery, Addis Abeba University, CHS. Consent from patients and their families was waived because the study was retrospective, data were anonymized, and most patients were deceased.

## Results

27 patients (14 males, 13 females) were identified over the 8-year study period, including 24 at TASH and 3 at MCM. Twenty-one were pediatric patients, aged from 1.5 to 15 years-old and six were adolescents or young adults older than 15 years old (**Table 1**). Median age at diagnosis was 10 years (range 1.5 to 23).

**Table 1:** Patients’ characteristics.

Nber	Sex & age (yr)	Preop_ PCPCS	M_staging	CSF Diversion	Interval diversion- resection	Preopsta y (days)	Intraop _resection	ICU stay (days)	PO complications	Histopath	PO ward stay (days)	PO PCPCS/KPS	PO Treatment	Reason for no PO Treatment	Recurre nce (months)	Survival (months)
1	F 1.5	3	NA			7	GTR	38	CSF leak, seizures, sepsis	DM	75	5		death (sepsis)		3
2	M 4	4	NA	VPS	50	21	NTR	2	Shunt malfunction & subdural hygroma	Classic	15	4		death (sepsis, shunt blockage, malnutrition)	1	3
3	F 5	4	NA			30	Biopsy	10	HAI	MBEN	10	5		death (sepsis)		1<
4	M 5	3	M2			1	GTR	3	Raised ICP & ventriculitis	MBEN	45	5		death (ventriculitis)		2
5	M 6	4	NA			8	NTR	4	ventriculitis	Classic	41	5		death (ventriculitis)		2
6	F 6	3	NA			10	GTR	3	brainstem dysfunction & morphine overdose	MBEN	3	5		death (brainstem injury and morphine overdose)		1<
7	M 6	4	M0			2	GTR	30	Raised ICP, PFM, transient lower CN palsy	Classic	30	4	CSI+CT			6 (shunt blockage)
8	M 6	3	NA	VPS	341	15	GTR	11	Raised ICP	Classic	40	4		death (raised ICP)		1
9	M 8	1	NA	ETV	36	18	GTR	107	bilateral CN VI palsy, HAI	Classic	0	5		death (sepsis)		1<
10	M 8	2	NA	ETV	102	22	STR	6	Hypertension & lower CN palsy	MBEN	1	5		death (sepsis)		1<
11	F 8	1	NA			1	NTR	50	ventriculitis & sepsis	Classic	49	5		death (ventriculitis)		2
12	M 8	3	M0	VPS	20	20	NTR	3	HAI	Classic	120	2	CSI+CT		26	36 (sepsis)
13	F 9	4	M0	VPS	29	30	GTR	2	HAP	Classic	180	3	Focal+CT		12	12 (infection)
14	F 10	1	NA			4	GTR	26	HAP, bilateral CN VI palsy, depressed gag & cough, raised ICP	Classic	30	3		death (brainstem dysfunction)		1
15	F 10	1	NA			3	STR[1st]&GTR[2nd]	45	Raised ICP & CN VI&VII palsy	Classic	30	2		death (sepsis)		1
16	M 11	2	NA			5	GTR	6	raised ICP [IVH]	DM	6	5		death (raised ICP)		1<
17	M 12	1	M2	ETV	279	40	GTR	13	ventriculitis, CN VI palsy	Classic	53	5		death (ventriculitis)		2
18	F 12	1	M0	ETV	21	6	STR	7	Raised ICP & pseudomeningocele	DM	60	2	CSI+CT		8	12 (sepsis)
19	M 12	1	NA			7	GTR	4	Raised ICP, pseudomeningocele & CSF leak	Classic	15	1	CSI			36+
20	F 14	3	NA			8	GTR	12	Brainstem death on 8th postop day	MBEN	12	5		death (brainstem death)		1<
21	F 15	2	NA			20	GTR	2		Classic	7	1	CSI			6 (LTFU)
22	M 17	60%	NA	VPS	18	18	STR	7		MBEN	15	60%		Treatment abandonment		12 (aspiration pneumonia)
23	F 18	905	M0	ETV	10	20	GTR	1		DM	7	80%	CSI		6	6 (disease)
24	M 19	80%	NA	ETV	7	15	GTR	1	Raised ICP, high EVD output & CSF leak	Classic	9	80%	CSI		4	6 (disease)
25	F 20	100%	NA			5	GTR	1		DM	7	100%		Treatment abandonment		6 (LTFU)
26	M 23	80%	NA	VPS	8	15	GTR	2		Classic	15	70%	Focal		36	36+
27	F 23	90%	M0			10	GTR	1	Acute Epidural Hematoma	DM	7	80%	CSI			6+ (LTFU)

M, male; F, female; PO, Postoperative; PCPCS, pediatric cerebral performance category scale; KPS, Karnofsky Performance Status; ETV, endoscopic third ventriculostomy; VPS, ventriculoperitoneal shunt; GTR, gross total resection; NTR, near total resection; STR, subtotal resection; ICP, intracranial pressure; CN, cranial nerve; PFM, posterior fossa mutism; IVH, intraventricular hemorrhage; CSF, cerebrospinal fluid; EVD, external ventricular drainage; HAI, hospital acquired infection; HAP, hospital acquired pneumonia; DM, desmoplastic; MBEN, medulloblastoma with extreme nodularity; CSI, craniospinal irradiation; CT, chemotherapy; LTFU, lost to follow-up.

The most prevalent presenting symptoms were headaches (23 patients), vomiting/nausea (21 patients) and balance disturbances (25 patients). Nine patients presented with cranial nerve deficits. Other common symptoms included nystagmus (4 patients), and seizures (4 patients). Twenty-two patients had mild-moderate functional disability at presentation. The recorded interval between the onset of symptoms and the diagnosis was 1 to 4 months, with 15 patients (55.6%) who presented within 3 months of their initial symptoms. Eleven pediatric patients presented with moderate to severe disability (Pediatric Cerebral Performance Category Scale 3 or 4, **Table 1**).

All study participants had a brain CT while brain MRI was done in 21 (77.8%) patients. Whole spine MRI was not done in 21 (77.8%) cases and only six patients (22.2%) underwent preoperative spinal imaging which did not reveal spinal seeding. No patients had a postoperative spinal MRI scan.

On radiologic evaluation, the tumor was originating from the vermis in 21 patients (77.8%) and from the cerebellar hemisphere in 6 patients. Twelve (44.4%) tumors measured more than 5 cm in largest diameter while smaller tumors measuring less than 3 cm were seen in only 2 (7.4%) patients. 5 cases (18.5%) demonstrated brainstem infiltration and majority (51.9%) had some degree of brainstem compression. Twenty-five (92.6%) patients had evidence of hydrocephalus on the preoperative imaging study.

The median preoperative stay on the neurosurgical ward was 10 days (range 1 to 40). The main reasons for longer preoperative stay were the unavailability of ICU beds and lack of access to operating rooms due to competing emergency patients. Twelve out of twenty-seven (44.4%) patients underwent preoperative CSF diversion. Of these patients, 6 underwent an endoscopic third ventriculostomy (ETV) and the remaining 6 underwent a ventriculo-peritoneal shunt (VPS) insertion. The median interval between CSF diversion and tumor resection was 25 days (range 7–341 days).

All patients underwent tumor resection. Surgery was performed for all in prone position and was performed under microscope. However, the Cavitron ultrasonic surgical aspirator (CUSA) and neuronavigation were not available. According to neurosurgical reports, 18 patients (66.7%) had intraoperative gross total resection (GTR), 4 a near total resection (NTR), 4 a subtotal resection (STR) and 1 a partial resection (PR). The extent of resection was confirmed on postoperative CT scan in 23 patients with 15 GTR, 3 NTR and 5 STR. Median duration of procedure was 6 hours (range 4 to 15). One patient had 2 resections. All patients were sent to ICU on the immediate postoperative course. Median duration of ICU stay was 6 days (range 1 to 107).

### Histology and staging

Histopathological diagnosis was made from hematoxylin and eosin-stained sections. There was no immunohistochemistry performed and no molecular studies, which were not available in either hospital Classic histology was the predominant finding in fifteen (55.6%) patients. Six patients (22.2%) had desmoplastic histology, and the remaining six patients (22.2%) had histological features described as medulloblastoma with extensive nodularity. There was no tumor with anaplastic histology.

Only 10 (37%) patients had a postoperative CSF examination that was negative in all. Overall, with only 6 MRI scans of the spine and 10 CSF examination, staging was incomplete in the majority of patients, with only 6 patients having a full staging. All these patients were non-metastatic, based on brain and spinal MRI and CSF results. As a result, full staging was unavailable for 21 patients. However, 2 patients had evidence of metastatic disease on the diagnostic brain MRI and were definitely high risk (M2) in this context.

### Postoperative course

Fourteen (51.9%) patients had a postoperative open external ventricular drainage (EVD). Twenty-two (81.5%) of the study participants had immediate postoperative complications. Ventriculitis was the most frequent complication noted in 4 patients, who all succumbed to infection. In patients who were conscious and assessable postoperatively, cerebellar mutism was seen in only one patient.

Fourteen (51.9%) patients required additional surgery for the management of hydrocephalus, ventriculitis and related complications. EVD and VPS each accounted for 43% of reoperations.

The duration of postoperative ward stay ranged from 0 to 180 days, with a median of 15 days. Among operated patients, a majority of pediatric patients (14/21), had severe-to-moribund disability (Lansky 0-40), whereas the majority of adolescent/adult patients (4/6), had a normal functional status.

Fourteen (51.8%) patients died before considering oncologic treatment. All were pediatric patients (14 out 21). The causes of death in these 14 patients were surgical mortality and perioperative infection: 6 patients from postoperative CSF leak, ventriculitis and sepsis, 3 from hospital acquired infection, 2 from brainstem dysautonomia, 2 from intraventricular hemorrhage and raised intracranial pressure and one patient from morphine overdose.

### Adjuvant therapy

Following surgery, patients were referred to the radiation oncology department at TASH. A total of 13 patients (7 children and 6 adolescents/adults) were referred for postoperative treatment. Three patients (2 adults and one child) did not receive any postoperative treatment, 2 adults due to abandonment and one child (patient 2, **Table 1**) due to low performance status. Ten patients received radiotherapy. Cobalt was used for radiation in all cases. Detailed reports on radiation techniques (dose per fraction, boost) were not available in the surgical records. Only 8 (29.6%) patients received craniospinal irradiation (CSI). Two (7.4%) patients had cranial RT only. Following radiotherapy, chemotherapy was offered as an option to patients and families. Only 4 (14.8%) pediatric patients received chemotherapy, all 4 after completing CSI (3 patients) or focal irradiation (1 patient).

The median interval duration between surgery and radiotherapy was 51.50 days (range 34 to 120) in pediatric patients and 60 days in adults. The mean craniospinal dose RT dose in pediatric patients was 32.17 Gy. (SD = 12.5) and 33.75Gy in adults. Seven (25.9%) patients had a post radio-chemotherapy scan, of which 3 (11.1%) showed residual abnormalities in keeping with residual tumor or recurrence or radiation necrosis.

**Figure 1:**
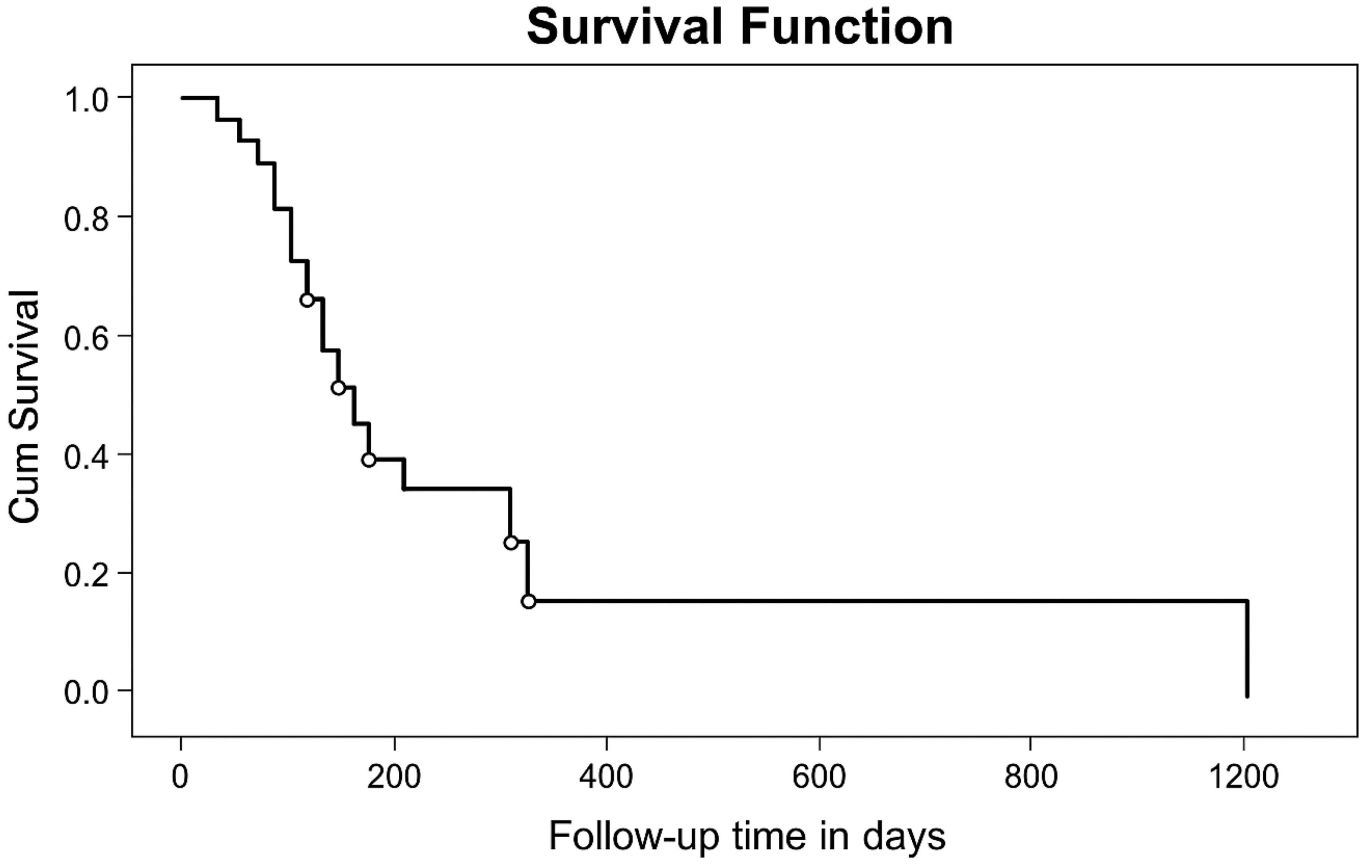
KM curve of overall survival probability of patients operated at TASH and MCM Hospitals, (n=27) from January 1, 2010, to April 30, 2018.

The 6-month and one-year OS probability was 41% and 29% with a median survival time of 107 days (95% CI: 49,166) for the whole group (**Figure 1**).

### Recurrence

Of the 10 patients referred to the oncology services (6 children, 4 adolescents/adults) and who received postoperative treatment, there were 5 recurrences. Recurrences occurred at a median interval of 9 months after diagnosis (range: 4 to 36 months). For this group of patients, overall 6-month PFS probability was 83% and one year PFS probability was 44%.

The pattern of relapse was local in all but one patient who suffered recurrence. The 2 patients who received focal radiotherapy to the posterior fossa relapsed. One was a child who experienced local recurrence at one year despite 10 cycles of adjuvant chemotherapy. The second was an adult patient who received focal radiotherapy only and showed spinal seeding 3 years later. Three children treated at the time of relapse died of chemotherapy complications.

### Survival

Upon subgroup analysis, 19 deaths were encountered amongst the 21 pediatric patients. From these, seven (33.33%) of the deaths occurred within the first post-operative month due to either surgical mortality or perioperative infections. Median survival time in pediatric patients was 66 days (95% CI: 27, 105), with 6 months and one-year OS rate of 24% (p=0.031) while in the adults, there were 2 documented deaths. Six-months and one-year OS probability in these adult patients were 100% and 67% (p=0.031). Two adult patients were lost to follow-up after their 6 months visit.

Because of the poor OS, statistical analysis to determine prognostic factors was limited. The presence of post-intervention complications had a statistically significant effect on survival outcome of patients in this series, with a median survival time of 382 days in patients without complications, versus 66 days when complications were recorded.

At one year, 5 patients (3 children, 2 adults) were alive and 4 attended their follow-up appointment. At a 3-year follow-up, there were 2 long-term survivors identified: one pediatric patient in complete remission with a Pediatric Cerebral Performance Category Scale (PCPCS) of 1 and one adult patient with spinal relapse and a Karnofsky performance status of 60%.

## Discussion

In this retrospective study, we identified 27 patients with medulloblastoma who underwent surgery in the 2 largest neurosurgical centers in Ethiopia. While all patients underwent resection, only 10 patients eventually received postoperative radiation and 4 proceeded to chemotherapy. These data reflect the gap between optimal management and a real-world experience in our setting.

In our study, the age at diagnosis was 10 years, with 77.8% of cases diagnosed were less than 15 years. Adolescents and adult patients accounted for 22.2% (n=6) of the study participants. This experience spans an 8-year period during which the expected number of pediatric medulloblastoma cases in a country of 120 million people with 40% age 15 years and less would have been at least 500 to 600, confirming that a large proportion of brain cancer cases in the pediatric population remains undiagnosed in low-income countries (7). The reasons for this enormous gap between the expected number of patients and our real-world data have not been investigated in this work. However, similar data on the low proportion of central nervous system tumors in children in low- and middle-income countries exist (8). Lack of awareness about central nervous system tumor and their presenting symptoms is a common source of delayed diagnosis (9). In LMIC, access to imaging studies is probably an additional obstacle, particularly when the cost of these studies is not covered by the health care system. Interestingly, the interval between initial symptoms and the diagnosis of posterior fossa tumor in our experience does not differ from data reported in high-income countries (10). However, the accuracy of these data in a retrospective chart review is always subject to questions. Efforts are ongoing to raise awareness about pediatric brain tumor in LMIC, and adapted programs based on the HeadSmart experience were recently tested in Jordan (11, 12).

From a surgical standpoint, most tumors were large in size, with 12/27 > 5cm in largest diameter and 13 between 3 and 5 cm. Using the Chang classification, 14 (51.9%) cases were staged “T4”. Brainstem compression was also evident in 14 (51.9%) patients. Evidence of raised increased cranial pressure (ICP) was present in 25 (92.6%) patients. Due to limitations in accessing the operating room and the lack of availability of ICU beds, delays to perform surgery were common and patients had to wait up to 40 days before tumor resection. Some patients underwent CSF diversion before radical surgery to control hydrocephalus. While this approach is not optimal, it is a common practice in LMIC, due to limited access to the operating room. The delay between CSF diversion and definitive surgery in this experience was 25 days, with the longest interval > 100 days in 3 pediatric patients who experienced immediate symptom relief after the intervention and as a result were not brought back by their parents until symptoms recurred. Surgery was done in prone position for all patients. The median duration of surgery was 6 hours, with a maximum of 15 hours. Data on the duration of medulloblastoma surgery are lacking for comparison purposes. In a series of 24 pediatric patients with medulloblastoma operated in La Paz, Lu et al. reported a mean operating time of 2.8 hours (13). However, in this La Paz series, there were no GTR, and most patients underwent STR (19/24) while 5 patients underwent biopsy only. The surgical material available in our setting is very limited compared to the equipment available in most operating rooms in high-income countries. In particular the lack of CUSA, a tool that has facilitated neurosurgical intervention for the resection of central nervous system tumor adjacent to critical structures (14). On paper, the resection rate achieved in our experience appears excellent with 15 GTR among 23 patients who had a postoperative CT scan. No patient had a postoperative assessment of resection by MRI scan, and it should be acknowledged that the use of postoperative CT is suboptimal for the assessment of residual tumor. Although the resection rate was high in this series, the poor postoperative status of the patients, particularly children, suggests that tumor resection was associated with major injuries to vital structures in several patients.

The rate of postoperative complications in our experience was high. Our data are in keeping with the experience reported in La Paz, with 13 of 27 patients who were discharged from hospital alive and referred to the oncology clinic in our experience, compared to 14/24 in the Bolivian series (13). High rates of perioperative mortality were also observed in a systematic review of surgical outcomes of pediatric brain tumors in Sub-Saharan Africa, where the one-year postoperative mortality ranged from 33 to 43% (15). In our cohort, the most common cause of death was infection, and in particular ventriculitis that was responsible for 4 early deaths. It is important to note that lack of appropriate material is a major issue for the postoperative care of neurosurgical patients. As an example, NG tubes are used instead of EVDs for ventricular drainage. This certainly explains the very high rate of infections in our series. Efforts are needed to improve perioperative care, including time to surgery, access to ICU, access to state-of-the-art surgical tools and materials, including CUSA, electrophysiological monitoring and neuronavigation.

The staging of our patients was very limited. Only 6 patients underwent spinal imaging, and 10 had CSF examination. Two patients were diagnosed with metastatic disease, but this is certainly an underestimate and integration of a proper staging for all newly diagnosed patients will be critical in the future for individualized and adapted treatment. The lack of systematic staging highlights the poor communication between the different actors involved in the management of children with brain tumors, Implementation of multidisciplinary meetings has shown benefits both in high and low-middle income countries, with a clear impact on decision-making and coordination of care (16).

Some of the patient factors impeding further administration of treatment following surgery were severe acute malnutrition (the mean BMI was 17 in this series), raised ICP, wound infection, ventriculitis, postoperative sepsis and irregular follow-up schedules. This suggests that patients present in poor general condition and as a result suffer from a high incidence of perioperative complications, a well-known issue in children with cancer in LMIC (17).

Only 8 patients underwent standard postoperative treatment with craniospinal irradiation, while 2 patients received focal radiotherapy to the posterior fossa. The median interval between surgery and initiation of radiotherapy was 51.5 days, with a range of 34 to 120 days, while most medulloblastoma protocols recommend initiation of radiotherapy within 4 to 6 weeks following surgery (1, 18). There was no possibility to review in detail radiation data, in particular radiation planning, rationale for doses and fractionation. As a result, the correlation between different potential factors influencing outcome (time of initiation, duration of radiation treatment, dose of craniospinal radiotherapy) is impossible. However, 2 patients received focal radiotherapy, and both experienced treatment failure. In the absence of proper staging for most patients and the lack of molecular studies and subgrouping, integration of modern approaches to allocate treatment – particularly radiation doses – is still an unrealistic objective. With the implementation of multidisciplinary meetings, we may hope that integration of the staging in the treatment plan will allow patients to receive a staged adapted management. Only 4 patients received adjuvant chemotherapy, which is a standard of care, at least in the pediatric population. These data clearly reflect the gap between LMIC and HIC, and the need to address disparities and inequalities regarding the global management of childhood cancer. Efforts are ongoing to develop guidelines for the management of various pediatric brain tumors, including medulloblastoma, according to local resources (19).

Our work has several limitations. The size of the cohort is small, and the data presented have therefore limited statistical power. The objective of this work was essentially descriptive, intending to compare the situation with future reports.

Despite these limitations, our study provides important insights regarding the specific surgical challenges in LMIC that are often overlooked in HIC. Duration of surgical ICU stays and postoperative EVDs were determining factors for survival in our study. These factors are not described in most literature. In our series, 7 patients experienced surgical mortality within one month, and 13 patients died within 2 months of surgery. All postoperative deaths occurred in patients </= 15 years old and accounted for 62% of pediatric deaths, implying majority of mortalities were due to surgical causes and perioperative infections like ventriculitis and sepsis. Most of the HIC literature suggests that the majority of deaths in operated patients are due to recurrence and metastasis (20, 21). This experience illustrates the critical need to improve operative and postoperative services to address the survival gap in neuro-oncology, particularly pediatric neuro-oncology, between LMIC and HIC.

## Conclusion

This experience provides a baseline assessment regarding the management of medulloblastoma in Ethiopia. While our results are disappointing, reflecting major gaps in the care of these complex patients, this review offers the opportunity to identify the steps needed to improve survival. Early diagnosis, timely management, appropriate risk stratification and standardized treatment with safe maximal resection and conventional CSI with combined chemotherapy are prerequisites for optimal survival rates both in children and adult patients. Efforts should also be made as well to implement radiation-sparing strategies for infants and young children (22). A recent report on the pediatric neuro-oncology activity at Tikur Anbessa Specialized Hospital between 2021 and 2024 suggests that there are now more referrals (69 patients with medulloblastoma and other embryonal tumors) along with an improvement in surgical outcomes and multidisciplinary care (23).

## Data Availability

The original contributions presented in the study are included in the article/supplementary material. Further inquiries can be directed to the corresponding author.
